# AKR1B1 and AKR1B10 as Prognostic Biomarkers of Endometrioid Endometrial Carcinomas

**DOI:** 10.3390/cancers13143398

**Published:** 2021-07-07

**Authors:** Marko Hojnik, Snježana Frković Grazio, Ivan Verdenik, Tea Lanišnik Rižner

**Affiliations:** 1 Institute of Biochemistry, Faculty of Medicine, University of Ljubljana, 1000 Ljubljana, Slovenia; marko.hojnik@mf.uni-lj.si; 2 Division of Gynecology, Department of Pathology, University Medical Centre Ljubljana, 1000 Ljubljana, Slovenia; snjezana.frkovicgrazio@kclj.si; 3 Division of Gynecology, Department of Obstetrics and Gynecology, University Medical Centre Ljubljana, 1000 Ljubljana, Slovenia; ivan.verdenik@guest.arnes.si

**Keywords:** endometrial carcinoma, survival, prognosis, immunohistochemistry, biomarker, aldo-keto reductase family 1 member B1 (AKR1B1), aldo-keto reductase family 1 member B10 (AKR1B10)

## Abstract

**Simple Summary:**

We evaluated the potential of AKR1B1 and AKR1B10 as tissue biomarkers of endometrial cancer by assessing the immunohistochemical levels of AKR1B1 and AKR1B10 in tissue paraffin sections from 101 well-characterized patients with endometrioid endometrial cancer and 12 patients with serous endometrial cancer. Significantly higher immunohistochemical levels of AKR1B1 and AKR1B10 were found in adjacent non-neoplastic endometrial tissue compared to endometrioid endometrial cancer. The group of patients with both AKR1B1 and AKR1B10 staining above the median values showed significantly better overall and disease-free survival compared to all other patients. Multivariant Cox analysis recognized a strong AKR1B1 and AKR1B10 staining as a statistically important survival prediction factor in patients with endometrioid endometrial cancer. In contrast, we observed no significant differences in AKR1B1 and AKR1B10 staining in patients with serous endometrial cancer. Our results suggest that AKR1B1 and AKR1B10 have protective roles in endometrioid endometrial cancer and represent prognostic biomarker candidates.

**Abstract:**

The roles of aldo-keto reductase family 1 member B1 (AKR1B1) and B10 (AKR1B10) in the pathogenesis of many cancers have been widely reported but only briefly studied in endometrial cancer. To clarify the potential of AKR1B1 and AKR1B10 as tissue biomarkers of endometrial cancer, we evaluated the immunohistochemical levels of AKR1B1 and AKR1B10 in tissue paraffin sections from 101 well-characterized patients with endometrioid endometrial cancer and 12 patients with serous endometrial cancer and compared them with the clinicopathological data. Significantly higher immunohistochemical levels of AKR1B1 and AKR1B10 were found in adjacent non-neoplastic endometrial tissue compared to endometrioid endometrial cancer. A trend for better survival was observed in patients with higher immunohistochemical AKR1B1 and AKR1B10 levels. However, no statistically significant differences in overall survival or disease-free survival were observed when AKR1B1 or AKR1B10 were examined individually in endometrioid endometrial cancer. However, analysis of AKR1B1 and AKR1B10 together revealed significantly better overall and disease-free survival in patients with both AKR1B1 and AKR1B10 staining above the median values compared to all other patients. Multivariant Cox analysis identified strong AKR1B1 and AKR1B10 staining as a statistically important survival prediction factor. Conversely, no significant differences were found in serous endometrial cancer. Our results suggest that AKR1B1 and AKR1B10 play protective roles in endometrioid endometrial cancer and show potential as prognostic biomarkers.

## 1. Introduction

Endometrial cancer (EC) is the most common gynecological cancer diagnosis in developed countries [1]. The prevalence of EC, which is predominantly found in postmenopausal women, is expected to increase in developed countries due to population aging [2] and the obesity epidemic [3,4].

EC is historically divided into two subgroups: type 1 includes estrogen-dependent carcinomas (80%) and type 2 includes estrogen-independent and more aggressive carcinomas (20%) [5]. The histopathological subtypes of EC include endometrioid, serous, clear cell, carcinosarcoma, mucinous, mixed cell, dedifferentiated, undifferentiated, and neuroendocrine carcinoma. The molecular classification of EC, proposed by The Cancer Genome Atlas (TCGA) Research Network, separates EC into four molecular prognostic groups: polymerase epsilon (POLE) ultramutated, microsatellite instability (MSI) hypermutated, copy-number-low/p53-wild-type (p53 wt), and copy-number-high/p53-mutated (p53mt) [6]. According to meta-analyses, this molecular classification is important for predicting prognoses and directing treatment strategies [6,7].

Traditionally, the precise surgical staging of EC requires removing the uterus, cervix, adnexa, and pelvic and para-aortic lymph nodes, obtaining pelvic washings, and performing pathological examinations. This enables establishing a precise diagnosis, identifying the extent of the disease, predicting the prognosis, and prescribing adjuvant radiation therapy and/or chemotherapy [1]. Patients with high-risk disease have a higher frequency of para-aortic lymph node metastases, suggesting that para-aortic lymphadenectomy should be performed as a part of the surgical staging in these patients. Since 2014, sentinel lymph node mapping has been incorporated into the management of EC as a means to evaluate the lymph nodes most likely to be involved in metastatic cancer and to limit the extent of surgery and lymphadenectomy [1]. Sentinel lymph node mapping has a good diagnostic accuracy and can safely replace lymphadenectomy in the staging of EC; however, its prognostic potential regarding high-risk EC remains to be evaluated [8,9,10].

Diagnosing EC is straightforward in most cases. However, sometimes, the poor reproducibility of the histological type and grade classification hinders the prediction of prognosis and selection of optimal treatment. New biomarkers may provide a more accurate and prognostically relevant approach for classifying these tumors.

The enzymes of the aldo-keto reductase (AKR) superfamily are involved in a plethora of important biochemical processes. The human members of AKR1B subfamily AKR1B1 and AKR1B10 catalyze NADPH-dependent reductions of carbonyl groups to hydroxyl groups and act on different endogenous and exogenous substrates. AKR1B1 catalyzes the reduction of glucose to sorbitol and plays a role in the polyol pathway, osmoregulation [11], prostaglandin PGF2α synthesis, and the protein kinase C pathway, which stimulates nuclear factor kappa B, inflammation, and proliferation [11,12,13,14]. AKR1B10 catalyzes the reduction of retinals to retinols, which depletes retinoic acid and leads to important pro-differentiating effects [15]. AKR1B10 is involved in the prenylation of small guanine nucleotide triphosphatases (GTPases) by reducing isoprenyl aldehydes and is thus implicated in cell proliferation [16]. AKR1B10 also regulates fatty acid biosynthesis, which plays an important role in carcinogenesis [17,18]. AKR1B1 and AKR1B10 confer resistance to numerous chemotherapeutics, including daunorubicin, idarubicin, and cisplatin [19,20]. Additionally, both AKR1B1 and AKR1B10 can exert protective actions and detoxify products of lipid peroxidation, e.g., convert cytotoxic carbonyls such as 4-hydroxynonenal to 4-hydroxynonenol [21].

In a limited number of EC samples, we previously observed a down-regulation of AKR1B1 mRNA and protein levels and up-regulation of AKR1B10 mRNA and down-regulation of AKR1B10 protein levels, compared to adjacent nontumor tissues [22]. In high-grade tumors, we observed significantly decreased ratios of AKR1B10 mRNA levels in tumor tissues versus adjacent control tissues [23,24,25,26,27]. To clarify the potential of AKR1B1 and AKR1B10 as tissue biomarkers of EC, we here immunohistochemically evaluated AKR1B1 and AKR1B10 levels in tissue paraffin sections from a larger group of well-characterized patients with EC and a small group of patients with serous EC.

## 2. Materials and Methods

### 2.1. Study Groups

This retrospective study included 113 patients with EC, 101 with endometrioid EC and 12 with serous EC, who were diagnosed form 2003 and 2014 and have been enrolled in our previous studies [22,28,29]. Paraffin-embedded tissue samples of the primary tumors were examined, and clinical and histopathological data were collected (Table 1) Tables S1–S5).

We evaluated the immunohistochemical (IHC) staining of AKR1B1 and AKR1B10 in EC. The results were compared with adjacent non-neoplastic endometrial tissue from the same patients when this tissue was available (*n =* 70). We examined the correlations with the other clinicopathological data, including overall survival, disease-free survival, stage of the disease, lymphovascular invasion, parous status, menopausal status, and smoking.

### 2.2. Immunohistochemistry

Immunohistochemistry was performed to visualize specific antigens on formalin-fixed, paraffin-embedded tissue samples of EC. All histopathological data and samples were collected from the archives of the University Medical Centre Ljubljana, Division of Gynecology, Department of Pathology, and were revised before inclusion in this study.

From each paraffin-embedded tissue block, 3–5 µm thick paraffin sections were cut and placed onto glass slides (Superfrost Plus; Thermo Scientific, Leicestershire, UK). The sections were dehydrated in a ventilation slide-drying oven for 60 min at 60 °C. IHC staining for AKR1B1 and AKR1B10 was carried out with an automatic system (BenchMark Ultra, Ventana, Basel, Switzerland) using detection kits (OptiView DAB; Ventana; Basel, Switzerland; cat. no. 760-700), following the manufacturer’s instructions.

Histological sections were deparaffinized (EZPrep solution; Ventana, Basel, Switzerland; cat. no. 950-102) for 4 min at 72 °C. Epitope retrieval was achieved using a Tris-based buffer, pH 8.5, for 24 min (for AKR1B10) and 32 min (for AKR1B1), at 95 °C (cell conditioning solution CC1; Ventana, Basel, Switzerland; cat. no. 950-124). The slides were incubated with the primary antibodies anti-AKR1B1 (Abcam, Cambridge, UK; cat. no. ab62795, lot: GR64780-2) and anti-AKR1B10 (Abcam, Cambridge, UK; cat. no. ab96417, lot: GR13314-31) for 32 min (diluted 1:200 in Antibody Diluent, Dako, Agilent, Santa Clara, USA, cat. no. S080983-2). The positive and negative controls for AKR1B1 and AKR1B10 were normal liver tissue (hepatocytes, ductal epithelium, and connective tissue), high-grade serous ovarian cancer (cancer cells, stroma), and uterine tissue (see validation of antibodies in Table 2). The assessment was conducted in blinded conditions and was based on the percentage of stained cells in the whole tumor area. Cytoplasmic and nuclear staining was considered as positive reaction. The stained tissue sections were evaluated by the author M.H.

### 2.3. Statistics

Student’s t-tests were used to compare the mean expressions of AKR1B1 and AKR1B10 (as percentages of positive cancer cells) between EC and adjacent non-neoplastic endometrial tissue. Linear regression analysis was performed to evaluate the correlations between AKR1B1 and AKR1B10 IHC expression and weight, height, and body mass index. The Kaplan–Meier method and Cox regression analysis were performed to evaluate survival. The Mann–Whitney U and Kruskal–Wallis tests were used to test for correlations between percentages of AKR1B1 and AKR1B10 expression and the other clinical data. All analyses were executed using SPSS software (IBM version 22, Armonk, NY, USA).

### 2.4. Ethical Issues

This retrospective study was approved by the National Medical Ethics Committee of the Republic of Slovenia (0120-701/2017-6).

## 3. Results

### 3.1. Demographic and Histopathological Characteristics of Patients

The study group (*n =* 113) included 101 endometrioid (65% grade I) and 12 serous endometrial carcinomas. Most patients (*n =* 96) were diagnosed with FIGO stage I disease (Table S1). The 5-year overall survival was 90.3%. Follow-up data were obtained for all 113 patients and ranged from 0.4 to 17.6 years (median = 8.6 years).

### 3.2. AKR1B1 and AKR1B10 Expression Levels in Endometrioid and Serous EC

**Figure 1:**
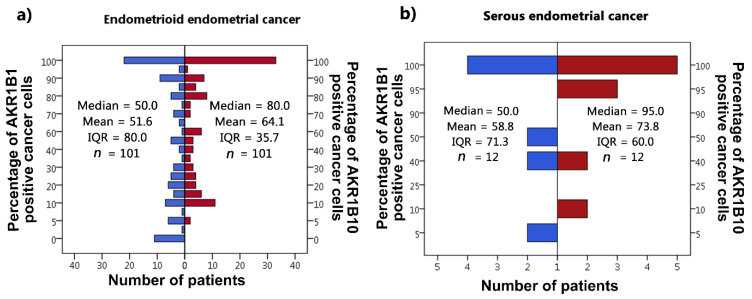
AKR1B1 and AKR1B10 immunohistochemical staining. The percentage of positive cancer cells is shown for (**a**) endometrioid EC and (**b**) serous EC. AKR1B1: aldo-keto reductase family 1 member B1; AKR1B10: aldo-keto reductase family 1 member B10; IQR: interquartile range; N: number of patients.

**Figure 2:**
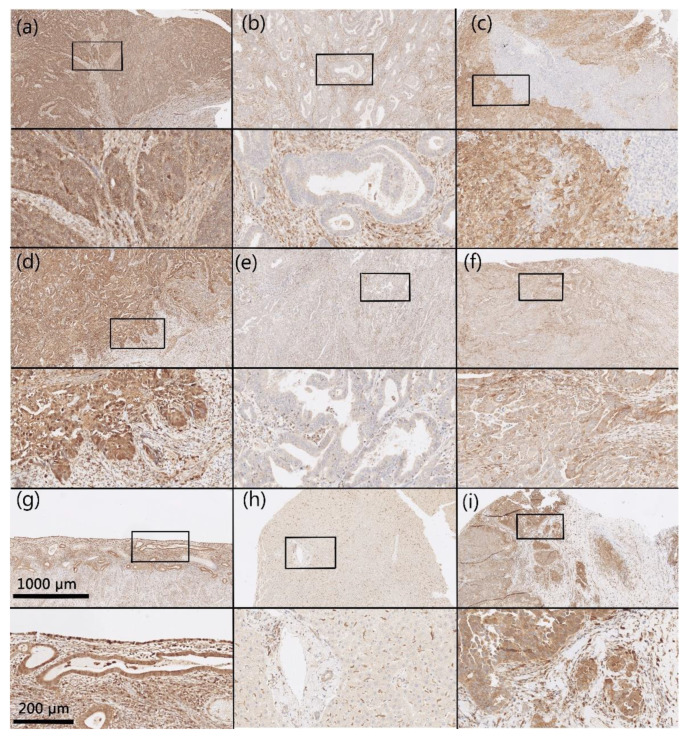
Representative immunohistochemical staining for AKR1B1. Samples of endometrioid EC (**a**–**c**), serous EC (**d**–**f**), non-neoplastic endometrial tissue (**g**), control liver tissue (**h**) and control high-grade serous ovarian cancer (**i**). Upper half of panels: 50× magnification; lower half of panels: the framed area from the upper half of the panel (200× magnification). Hematoxylin and eosin (HE) stained sections (Figure S1).

**Figure 3:**
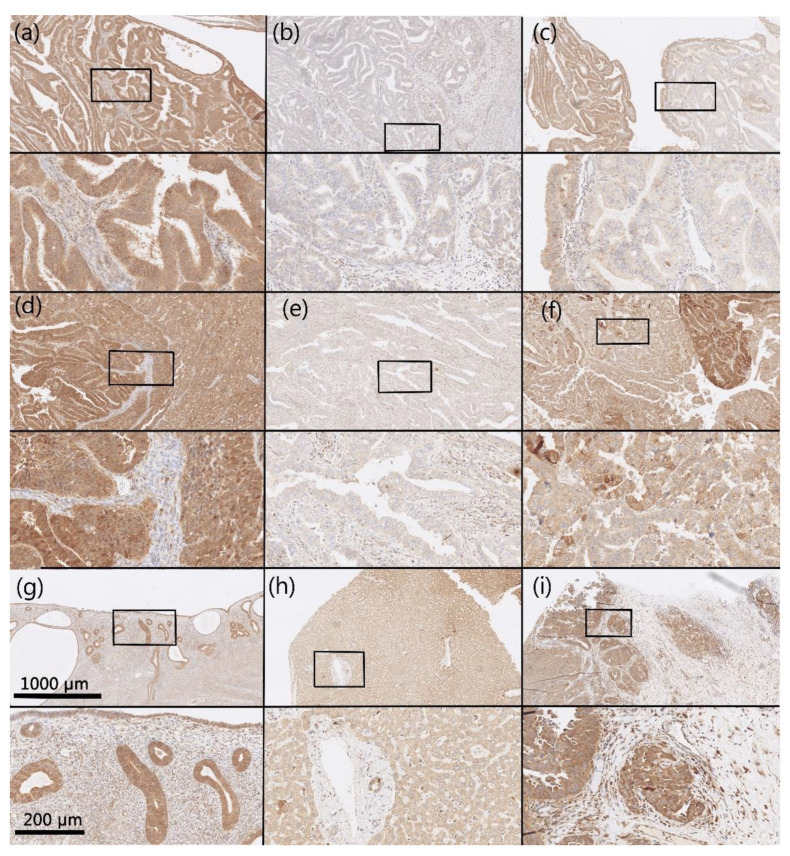
Representative immunohistochemical staining for AKR1B10. Samples of endometrioid EC (**a**–**c**), serous EC (**d**–**f**), non-neoplastic endometrial tissue (**g**), control liver tissue (**h**), and control high-grade serous ovarian cancer (**i**). Upper half of panels: 50× magnification; lower half of panels: the framed area from the upper half of the panel (200× magnification). Hematoxylin and eosin (HE) stained sections (Figure S2).

IHC reactions were positive for AKR1B1 and AKR1B10 within the cytoplasm and nucleus of the epithelial cancer cells in both endometrioid and serous EC as well as in the endothelium. Positive cytoplasmic and nuclear staining of tumor cells was calculated as percentages. In all cases in which positive reaction cytoplasmic staining was seen, out of those cases, some cases also had positive nuclear reaction; in most cases, nuclear staining was weaker than cytoplasmic staining. The median and mean percentages of AKR1B1-positive cancer cells were 50.0% and 51.6% for endometrioid EC and 50.0% and 58.8% for serous EC. The median and mean percentages of AKR1B10-positive cancer cells were 80.0% and 64.1% for endometrioid EC and 95.0% and 73.8% for serous EC (Figure 1). Adjacent non-neoplastic endometrial tissue revealed strong positive reactions within the nucleus and cytoplasm of endometrial glands, with median and mean percentages of AKR1B1-positive epithelial cells of 100.0% and 90.0% in patients with endometrioid EC and 100.0% and 88.3% in patients with serous EC; the myometrium and endometrial stroma were negative (Figure 2). The median and mean percentages of AKR1B10-positive epithelial cells in adjacent non-neoplastic endometrial tissue were 100.0% and 94.1% in patients with endometrioid EC and 100% and 94.4% in patients with serous EC; the myometrium and endometrial stroma were negative (Figure 3).

### 3.3. Comparison of AKR1B1 and AKR1B10 Expression Levels in Endometrioid and Serous EC and Adjacent Non-Neoplastic Endometrial Tissue

**Figure 4:**
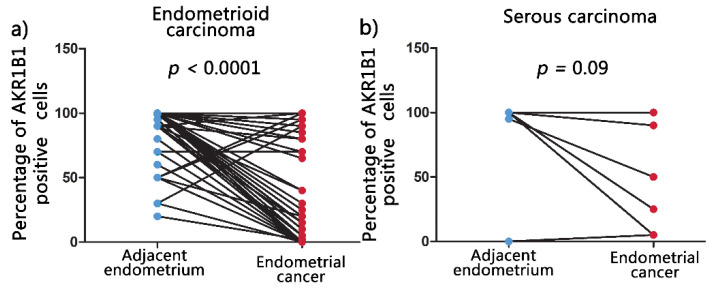
AKR1B1 immunohistochemical expression in EC and the adjacent non-neoplastic endometrial tissue. Before and after graphs show the expression of AKR1B1 in endometrial cancer tissue and its paired adjacent non-neoplastic endometrial tissue. (**a**) Adjacent non-neoplastic endometrial tissue (*n =* 61, mean = 90.1, median = 100, IQR = 10) compared to endometrioid EC (*n =* 61, mean = 55.8, median = 70.0, IQR = 90.0). (**b**) Adjacent non-neoplastic endometrial tissue (*n =* 9, mean = 88.3, median = 100, IQR = 2.5) compared to serous EC (*n =* 9, mean = 63.9, median = 90.0, IQR = 85.0). AKR1B1: aldo-keto reductase family 1 member B1; IQR: interquartile range; *N*: number of patients.

Non-neoplastic endometrial tissue adjacent to the carcinomas was available for IHC analysis in 70 out of the 113 cases. Significantly higher percentages (mean ± SD) of AKR1B1-positive epithelial cells were observed in adjacent non-neoplastic endometrial tissue (90.1% ± 20.3%) compared to endometrioid EC (55.8% ± 41.8%) (*p* < 0.0001; Figure 4a). No significant differences were observed between adjacent non-neoplastic endometrial tissue (88.3% ± 33.2%) and serous EC (63.9% ± 42.6%) (*p* = 0.09; Figure 4b).

**Figure 5:**
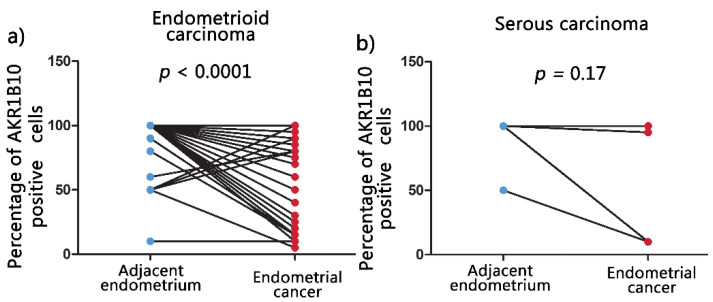
AKR1B10 immunohistochemical expression in EC and the adjacent non-neoplastic endometrial tissue. Before and after graphs show the expression of AKR1B1 in endometrial cancer tissue and its paired adjacent non-neoplastic endometrial tissue. (**a**) Adjacent non-neoplastic endometrial tissue (*n =* 61, mean = 94.1, median = 100, IQR = 0) compared to endometrioid EC (*n =* 61, mean = 65.5, median = 80.0, IQR = 75.0). (**b**) Adjacent non-neoplastic endometrial tissue (*n =* 9, mean = 94.4, median = 100, IQR = 0) compared to serous EC (*n =* 9, mean = 78.9, median = 100, IQR = 47.5). AKR1B10: aldo-keto reductase family 1 member B10; IQR: interquartile range; *N*: number of patients.

Similar results were observed for AKR1B10 staining. Significantly higher mean percentages of positive epithelial cells (mean ± SD) (94.1% ± 17.4%) were found in adjacent non-neoplastic endometrial tissue compared to endometrioid EC (65.5% ± 35.4%) (*p* < 0.0001; Figure 5a). Similarly, as with AKR1B1, also AKR1B10 staining did not show significant differences between adjacent non-neoplastic endometrial tissue (94.4% ± 16.7%) and serous EC (78.9% ± 39.1%) (*p* = 0.17; Figure 5b).

### 3.4. The Correlation of AKR1B1 and AKR1B10 Expression Levels with Survival

**Figure 6:**
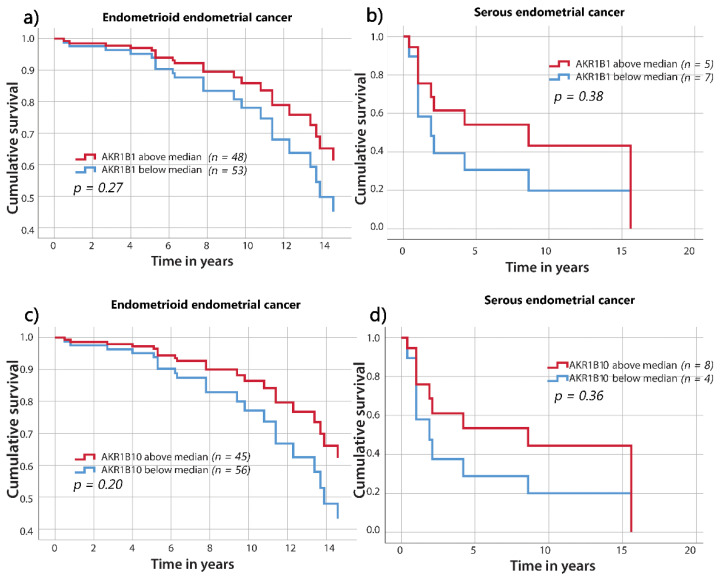
Overall survival curves for AKR1B1 and AKR1B10 in patients with endometrioid and serous EC. AKR1B1 in patients with (**a**) endometrioid EC (*p* = 0.27) and (**b**) serous EC (*p* = 0.38). AKR1B10 in patients with (**c**) endometrioid EC (*p =* 0.20) and (**d**) serous EC (*p* = 0.36). The groups are separated according to their median values. Time on *x*-axis represents time elapsed since initial diagnosis. AKR1B1: aldo-keto reductase family 1 member B1; AKR1B10: aldo-keto reductase family 1 member B10.

To evaluate AKR1B1 and AKR1B10 expression in relation to the clinicopathological data, endometrioid and serous EC were divided into two groups using the median values of AKR1B1 or AKR1B10 expression as the threshold values. The Kaplan–Meier and Cox survival models were used for the survival studies. As shown by the survival curves (Figure 6), there were no statistical differences in overall survival between the groups above and below the median percentages of AKR1B1- or AKR1B10-positive cancer cells in endometrioid or serous EC. However, there was a trend for a higher survival of patients with higher percentages of AKR1B1- and AKR1B10-positive cells. When groups were separated into tertiles, quartiles, and quintiles, no significant differences were observed.

**Figure 7:**
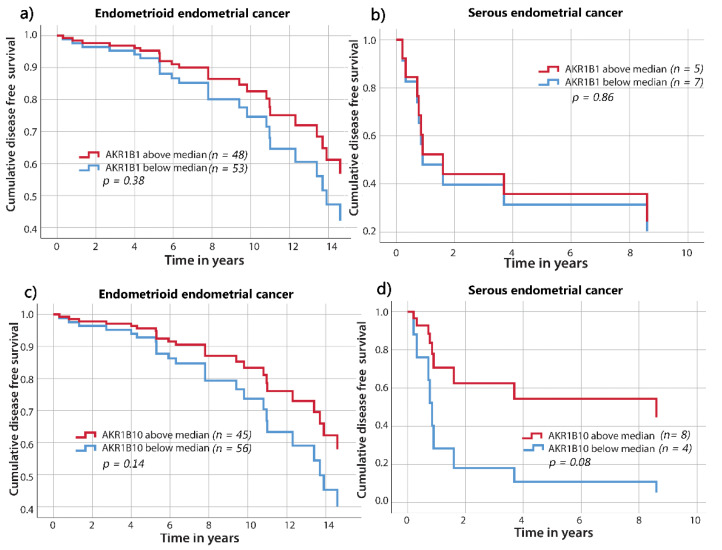
Disease-free survival curves for AKR1B1 and AKR1B10 in endometrioid and serous EC. AKR1B1 in patients with (**a**) endometrioid EC (*p =* 0.38) and (**b**) serous EC (*p =* 0.86). AKR1B10 in patients with (**c**) endometrioid EC (*p =* 0.14) and (**d**) serous EC (*p =* 0.08). The groups are separated according to their median values. Time on x axis represents time elapsed since initial diagnosis. AKR1B1: aldo-keto reductase family 1 member B1; AKR1B10: aldo-keto reductase family 1 member B10.

Similarly, the disease-free survival curves did not differ significantly between groups above and below the median percentages of AKR1B1- or AKR1B10-positive cancer cells in endometrioid or serous EC (Figure 7).

**Figure 8:**
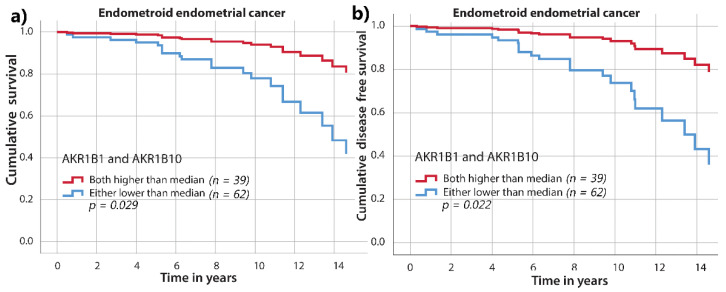
Overall survival and disease-free survival in relation to AKR1B1 and AKR1B10. One group includes cases with both AKR1B1 and AKR1B10 staining above the median values, and the other group includes cases with either AKR1B1 or AKR1B10 below the median values in endometrioid EC. (**a**) Overall survival (*p =* 0.029) and (**b**) disease-free survival curves (*p =* 0.022). Time on x axis represents time elapsed since initial diagnosis. AKR1B1: aldo-keto reductase family 1 member B1; AKR1B10: aldo-keto reductase family 1 member B10. Multivariant Cox analyses of independent prediction factors of overall and disease-free survival were performed (Table 3). For overall survival, the significant prediction factors, in descending order, were lymphovascular invasion (*p* = 0.001), risk group (EC grade I–II vs. EC III or SC) (*p =* 0.005), and AKR1B1 and AKR1B10 expression above the median values (*p* = 0.036). For disease-free survival, the significant prediction factors were lymphovascular invasion (*p* = 0.003), risk group (EC grade I–II vs. EC III or SC) (*p* = 0.004), and AKR1B1 and AKR1B10 expression above the median values (*p* = 0.023).

For endometrioid EC, we also performed survival analysis, which included IHC staining for both AKR1B1 and AKR1B10. Cases were stratified into two groups according to the median values of AKR1B1 and AKR1B10. The first group included cases with both AKR1B1 and AKR1B10 staining above the median. The second group included all other cases with either or both AKR1B1 and AKR1B10 staining below the median. We found significantly better overall survival (*p* = 0.029) and disease-free survival (*p* = 0.022) in the first group compared to the second group (Figure 8). In addition, when we limited analysis only to low-risk endometrioid EC (grade 1 and 2) and to low FIGO stage (FIGO I–II), we found significantly better overall survival (*p* = 0.023 and *p* = 0.020) and disease-free survival (*p* = 0.022 and *p* = 0.014) in the group with AKR1B1 and AKR1B10 staining above the median compared to group that included all other cases (Figures S3 and S4, Tables S6 and S7).

### 3.5. The Correlation of AKR1B1 and AKR1B10 Expression Levels with Other Clinical Data

No statistically significant correlations were found between the percentages of AKR1B1- or AKR1B10-positive cells and the FIGO stage (I–II or III–IV) for endometrioid EC (*p* = 0.96 for AKR1B1 and *p* = 0.83 for AKR1B10; Mann–Whitney U tests) or serous EC (*p* = 0.60 for AKR1B1 and *p* = 0.37 for AKR1B10; Mann–Whitney U tests). Grade III endometrioid EC compared to grade I–II did not show significantly different levels of AKR1B1 (*p* = 0.96) or AKR1B10 (*p* = 0.29) (Mann–Whitney U test). In patients with endometrioid and serous EC, there were no significant correlations between AKR1B1 or AKR1B10 expression and age, height, weight, body mass index, parous status, menopausal status, smoking status, invasion in lymph vessels, lymph nodes, or myometrium, or cervix or parametria (Chi-squared test, T-test analysis; Tables S8 and S9).

## 4. Discussion

In the present study, we assessed IHC AKR1B1 and AKR1B10 levels in a larger cohort of patients with endometrioid (*n =* 101) EC and, for the first time, in a small cohort of patients with serous (*n =* 12) EC and correlated AKR1B1 and AKR1B10 expression with clinicopathological data.

There is an emerging interest in studying the role of AKR in the pathogenesis of different pathologies [30], including uterine diseases [31,32]. Many studies demonstrated that AKR1B1 and AKR1B10 are involved in different cancers [12,27,33,34,35,36].

In some types of cancer, the published studies associated AKR1B1 and AKR1B10 with poor survival of patients, while in other types of cancer, the protective effects were seen. The overexpression of AKR1B1 has been reported in breast, ovarian, cervical, lung, hepatocellular, and rectal cancer [27,37,38,39,40], but down-regulation of AKR1B1 has been observed in adenocarcinoma samples compared to adjacent nontumor tissue in endometrial and colorectal cancer [22,24,25,26]. In gastric carcinoma, oral squamous cell and lung adenocarcinoma AKR1B10 overexpression was associated with significantly poorer prognosis [34,35,41,42], but AKR1B10 was downregulated in colorectal cancer cells compared to the adjacent normal colorectal tissues, and the survival of AKR1B10 negative patients was significantly worse [43].

So far, only three studies examined AKR1B1 or AKR1B10 expression in EC. Yoshitake et al. [44] reported that AKR1B10 is expressed in EC but found no correlation with the clinicopathological features [44]. The other two studies were performed by our group. We first examined AKR1B1 and AKR1B10 mRNA and protein expression in 47 patients with EC [22]. We found lower AKR1B1 mRNA and protein levels in cancerous tissue compared to adjacent control tissue. Furthermore, we found higher AKR1B10 mRNA levels but lower protein levels in cancerous tissue compared to adjacent control tissue, which might be explained by the different protein translation efficiencies in cancer tissues [22]. Further IHC analysis revealed positive staining for AKR1B1 and AKR1B10 in all 22 EC and adjacent control tissue samples [22]. In the second published study, we examined the ratio of AKR1B1 and AKR1B10 mRNA levels in tumor tissues versus adjacent control tissues in relation to the clinicopathological data of 51 patients [23]. We found that body weight and BMI were negatively correlated with AKR1B1 and positively correlated with AKR1B10. Furthermore, AKR1B10 mRNA levels were significantly lower in high-grade compared to low-grade EC. The same study also revealed that age, myometrial invasion, FIGO stage, and lymphovascular invasion were not associated with AKR1B1 or AKR1B10 expression [23].

These previous findings are not completely in line with the present study. As observed previously [22], results at the mRNA level do not always correlate with those at the protein level, which is possibly due to different factors that affect translation. At the protein level, we here confirmed our published Western blot analysis of 30 paired tissue samples [22], as we found higher AKR1B1 and AKR1B10 IHC levels in control endometrium versus adjacent cancerous tissue from 61 patients with endometrioid EC. In the current study, we thus demonstrated that AKR1B1 and AKR1B10 protein levels are significantly lower in endometrioid EC compared to adjacent control endometrial tissues, while no significant differences were observed in serous EC.

Although there was a trend of better survival in patients with higher AKR1B1 and AKR1B10 IHC levels, survival studies of endometrioid EC did not show significant differences in overall and disease-free survival when AKR1B1 and AKR1B10 were examined individually. However, the group with both AKR1B1 and AKR1B10 IHC levels above the median values had significantly better overall and disease-free survival compared to other patients. These results indicate that higher AKR1B1 and AKR1B10 levels in cancer tissues correlate with better prognoses of patients with endometrioid EC, suggesting a protective role of AKR1B1 and AKR1B10. Additionally, multivariant Cox analysis identified high AKR1B1 and AKR1B10 expression as an important prediction factor for both overall and disease-free survival.

AKR1B1 and AKR1B10 exert a plethora of physiological actions and are involved in prostaglandin synthesis, retinoid metabolism, prenylation, lipid synthesis, and detoxification of unsaturated carbonyl products of lipid peroxidation [23,31]. Thus, AKR1B1 and AKR1B10 can stimulate inflammation and proliferation and attenuate differentiation and apoptosis. Conversely, the protective role of AKR1B1 and AKR1B10 can be explained by their involvement in detoxifying different products (e.g., 4-hydroxynonenal) of lipid peroxidation, which is a consequence of oxidative stress [45]. Oxidative stress and electrophilic stress are recognized by nuclear erythroid 2-related factor 2 (NRF2), which is a master regulator of numerous antioxidant/detoxifying genes, such as the AKR genes *AKR1B1* and *AKR1B10*. NRF2 binds to and up-regulates the expression of the antioxidant response elements of these genes. This is supported by studies showing that NRF2 inducers elevate *AKR1B1* and *AKR1B10* expression and that NRF2 signaling is activated by chemicals that produce reactive oxygen species [46,47,48].

AKR1B1 and AKR1B10 play similar roles in detoxifying lipid peroxidation products but exhibit different catalytic efficiencies for reducing 4-hydroxynonenale [49]. In this study, better survival was observed for patients with high IHC levels (staining above the median values) of both AKR1B1 and AKR1B10. These high levels of AKR1B1 and AKR1B10 were also identified as significant predictive markers for both overall and disease-free survival. This implies that the combined action of these enzymes is needed to exert protective effects. Our data suggest that AKR1B1 and AKR1B10 are involved in the pathogenesis of endometrioid EC; however, their exact roles still need to be determined. In serous EC, no significant differences were found; however, due to a very limited number of cases (*n* = 12), these results should be considered with caution.

## 5. Conclusions

In summary, our results indicate that AKR1B1 and AKR1B10 may play protective roles in the pathogenesis of endometrioid EC and show prognostic potential, as high levels of both AKR1B1 and AKR1B10 predict better patient survival. These findings are important because examining AKR1B1 and AKR1B10 expression might improve prognostic accuracy and influence on treatment planning for patients with endometrioid EC. The importance of AKR1B1 and AKR1B10 was not confirmed for serous EC, which was most probably due to the limited number of cases. To clarify the complex roles of AKR1B1 and AKR1B10 in the pathogenesis of endometrioid and serous EC, further studies are needed.

## Figures and Tables

**Table 1:** Clinical and histopathological data of the patients with EC.

Characteristic	Detail	Datum
Age (y) (*n =* 113)	Mean ± SD	63.6 ± 10.1
Weight (kg) (*n =* 108) ^a^	Mean ± SD	82.4 ± 17.2
Height (cm) (*n =* 104) ^a^	Mean ± SD	162.0 ± 5.5
Body mass index (kg/m^2^) (*n =* 104) ^a^	Mean ± SD	31.5 ± 6.7
Menopausal status (*n =* 108) (n (%)) ^a^	Postmenopausal	94 (87.0)
Parity status (*n =* 106) (n (%)) ^a^	Multiparous	93 (87.7)
Smoking status (*n =* 59) (n (%)) ^a^	Smokers	5 (8.5)
Histological type (*n =* 113) (n (%))	Endometrioid	101 (84.6)
	Serous	12 (9.8)
Histological grade (*n =* 101) (n (%))	G1	65 (62.5)
	G2	25 (24.0)
	G3	11 (10.6)
Myometrial invasion (*n =* 113) (n (%))	<50%	84 (74.3)
	≥50%	29 (25.7)
Lymphovascular invasion (*n =* 113) (n (%))		30 (26.5)
FIGO stage (*n =* 108) (n (%)) ^a^	I–II	97 (89.8)
	III–IV	11 (10.1)
Surgical resection (*n =* 113) (n (%))	R0	108 (95.6)
Lymphadenectomy (*n =* 113) (n (%))		105 (92.9)
Adjuvant chemotherapy (*n =* 113) (n (%))	Paclitaxel/Carboplatin	7 (6.2)
	Carboplatin	1 (0.1)
Adjuvant radiotherapy (*n =* 113) (n (%))		36 (31.9)
Overall survival (*n =* 113) (y)		
	Range	0.4–17.6
	Median	7.6
Disease-free survival (*n =* 113) (y)		
	Range	0.2–17.6
	Median	6.95

^a^ Cases with missing data. N: number of patients; y: years elapsed since initial diagnosis; SD: standard deviation.

**Table 2:** Antibody description and validation.

Antibody Information					
Antibody	Manufacturer, Catalogue Number, Lot Number	Peptide/Protein Target	Antigen Sequence	Species Raised, Monoclonal, Polyclonal	Dilution
Anti-AKR1B1	Abcam, Cambridge, UK, ab62795,GR64780-2	Aldo-keto reductase family 1 member B1	aa 300 to the C-terminus (conjugated to keyhole limpet hemocyanin)	Polyclonal rabbit antibody	1:200
Anti-AKR1B10	Abcam, Cambridge, UK, ab96417,GR13314-31	Aldo-keto reductase family 1 member B10	fragment corresponding to aa 1-286	Polyclonal rabbit antibody	1:200
Antibody Validation					
Published validation by our research team [22]					
Current Validation Positive controls for AKR1B1: Kupffer cells, lymphocytes, high-grade serous ovarian cancer cells Positive controls for AKR1B10: Hepatocytes, ductal liver epithelium, lymphocytes, high-grade serous ovarian cancer cells Negative controls for AKR1B1: hepatocytes, fibrous tissue Negative controls for AKR1B10: fibrous tissue					

AKR1B1: aldo-keto reductase family 1 member B1; AKR1B10: aldo-keto reductase family 1 member B10.

**Table 3:** Data on multivariant analysis of independent factors predictive of survival.

Overall Survival	Significance	Hazard Ratio	Confidence Interval
Lymphovascular invasion	*p* = 0.001	3.8	1.8–8.2
Risk group (EC grade I–II vs. EC III or SC)	*p* = 0.005	2.9	1.4–6.2
AKR1B1 and AKR1B10 expression above the median values	*p* = 0.036	0.4	0.1–0.9
FIGO (I–II vs. III–IV)	*p* = 0.103	2.2	0.9–5.5
Disease-free survival			
Lymphovascular invasion	*p* = 0.003	3.2	1.5–6.7
Risk group (EC grade I–II vs. EC III or SC)	*p* = 0.004	2.9	1.4–6.1
AKR1B1 and AKR1B10 expression above the median values	*p* = 0.023	0.3	0.1–0.9
FIGO (I–II vs. III–IV)	*p* = 0.126	2.0	0.8–5.0

## Data Availability

The data presented in this study are available in supplementary material.

## Supplementary Materials

The following are available online at https://www.mdpi.com/article/10.3390/cancers13143398/s1. Table S1: IHC data and tumor data; Table S2: Survival data; Table S3: Pathological data; Table S4: Clinical data 1; Table S5: Clinical data 2; Table S6: Survival analysis for low/high-risk endometrioid EC; Table S7: Survival analysis for FIGO I–II stage and for FIGO III–IV EC; Table S8: Associations between clinical and histopathological data and AKR1B1 and AKR1B10 IHC levels in patients with EC—Part 1; Table S9: Associations between clinical and histopathological data and AKR1B1 and AKR1B10 IHC levels in patients with EC—Part 2; Figure S1: Representative hematoxylin and eosin (HE) stained sections of the same samples as shown in Figure 2 in the main text of the manuscript; Figure S2: Representative hematoxylin and eosin (HE) stained sections of the same samples as shown in Figure 3 in the main text of the manuscript, Figure S3: Overall survival and disease-free survival in relation to AKR1B1 and AKR1B10 for patients with low/high-risk endometrioid EC. Figure S4: Overall survival and disease-free survival in relation to AKR1B1 and AKR1B10 for patients with FIGO I–II/FIGO III–IV disease.
