# Supplementary material for: AKR1B1 and AKR1B10 as Prognostic Biomarkers of Endometrioid Endometrial Carcinomas
Source: Cancers (Basel). 2021 Jul 7;13(14):3398. doi: 10.3390/cancers13143398 (PMC8305663; doi:10.3390/cancers13143398)
Supplement: Supplementary file 1 [file cancers-13-03398-s001.zip › cancers-1251396-supplementary.pdf]

cancers

MDPI

AKR1B1 and AKR1B10 as Prognostic Biomarkers of Endometrioid Endometrial Carcinomas

Marko Hojnik [1], Snježana Frković Grazio [2], Ivan Verdenik [3] and Tea Lanišnik Rižner [1,*]

Supplement Table S1. IHC data and tumor data

Case	Carcinoma type	AKR1B1 tumor*	AKR1B10 tumor*	AKR1B1 adjacent*	AKR1B10 adjacent*	Grade**	FIGO stage
**1**	ec	25.0	35.0	NA	NA	1	NA
**2**	ec	30.0	70.0	100.0	100	1	IA
**3**	ec	10.0	60.0	NA	NA	1	NA
**4**	ec	100.0	100.0	100.0	100.0	1	IA
**5**	ec	100.0	100.0	100.0	100.0	1	IA
**6**	ec	65.0	100.0	100.0	100.0	2	IB
**7**	ec	7.0	15.0	NA	NA	1	IV
**8**	ec	2.0	90.0	20.0	100.0	1	IB
**9**	ec	20.0	40.0	NA	NA	2	NA
**10**	ec	20.0	100.0	NA	NA	1	IA
**11**	ec	.0	60.0	NA	NA	1	IB
**12**	ec	65.0	100.0	NA	NA	1	IA
**13**	ec	5.0	20.0	NA	NA	2	IA
**14**	ec	25.0	10.0	NA	NA	2	IA
**15**	ec	95.0	100.0	100.0	100.0	1	IA
**16**	ec	75.0	90.0	NA	NA	1	IA
**17**	ec	5.0	50.0	100.0	100.0	2	IB
**18**	ec	40.0	10.0	100.0	100.0	3	IA
**19**	ec	40.0	40.0	90.0	100.0	1	IA
**20**	ec	10.0	10.0	100.0	100.0	1	IA
**21**	ec	10.0	20.0	NA	NA	3	IB

**22**	ec	70.0	100.0	NA	NA	3	IB
**23**	ec	100.0	100.0	100.0	50.0	1	IA
**24**	ec	90.0	100.0	100.0	100.0	1	IA
**25**	ec	100.0	60.0	100.0	100.0	2	IA
**26**	ec	90.0	100.0	NA	NA	1	IA
**27**	ec	80.0	80.0	90.0	100.0	2	IA
**28**	ec	70.0	75.0	100.0	100.0	2	IIIA
**29**	ec	95.0	85.0	50.0	100.0	1	IA
**30**	ec	15.0	90.0	100.0	100.0	1	IA
**31**	ec	50.0	30.0	NA	NA	2	IA
**32**	ec	30.0	90.0	NA	NA	2	IA
**33**	ec	100.0	100.0	100.0	100.0	3	IA
**34**	ec	20.0	50.0	100.0	100.0	1	IA
**35**	ec	80.0	100.0	NA	NA	1	IA
**36**	ec	90.0	100.0	30.0	100.0	2	IA
**37**	ec	70.0	50.0	70.0	100.0	1	IA
**38**	ec	100.0	100.0	100.0	100.0	1	IA
**39**	ec	85.0	70.0	100.0	100.0	1	IA
**40**	ec	5.0	15.0	100.0	100.0	1	IA
**41**	ec	5.0	80.0	NA	NA	1	IA
**42**	ec	50.0	100.0	NA	NA	1	IA
**43**	ec	50.0	100.0	NA	NA	1	II
**44**	ec	35.0	100.0	NA	NA	1	IA
**45**	ec	15.0	25.0	90.0	100.0	3	IB
**46**	ec	.0	60.0	90.0	100.0	1	IA
**47**	ec	10.0	95.0	100.0	100.0	1	IA
**48**	ec	20.0	80.0	50.0	50.0	2	IB
**49**	ec	50.0	100.0	NA	NA	1	IA

**50**	ec	.0	80.0	80.0	100.0	2	IA
**51**	ec	20.0	60.0	NA	NA	1	IA
**52**	ec	.0	10.0	70.0	100.0	2	IB
**53**	ec	.0	10.0	95.0	100.0	1	IA
**54**	ec	.0	40.0	NA	NA	1	IA
**55**	ec	20.0	30.0	NA	NA	2	IA
**56**	ec	15.0	15.0	99.0	100.0	1	IA
**57**	ec	.0	80.0	100.0	60.0	1	IA
**58**	ec	.0	15.0	60.0	80.0	1	IA
**59**	ec	25.0	100.0	100.0	100.0	2	IIIC
**60**	ec	80.0	100.0	100.0	100.0	1	IA
**61**	ec	100.0	100.0	100.0	100.0	1	IA
**62**	ec	70.0	100.0	NA	NA	1	IA
**63**	ec	5.0	5.0	NA	NA	2	IB
**64**	ec	50.0	100.0	NA	NA	1	IB
**65**	ec	.0	20.0	NA	NA	2	IA
**66**	ec	10.0	80.0	NA	NA	3	IIIC
**67**	ec	100.0	100.0	50.0	100.0	1	IA
**68**	ec	100.0	100.0	100.0	100.0	3	IB
**69**	ec	10.0	60.0	100.0	100.0	2	IA
**70**	ec	60.0	90.0	NA	NA	1	IA
**71**	ec	.0	75.0	50.0	100.0	3	IA
**72**	ec	100.0	100.0	NA	NA	1	IA
**73**	ec	100.0	100.0	100.0	100.0	1	IA
**74**	ec	.0	15.0	30.0	90.0	1	IA
**75**	ec	10.0	80.0	100.0	100.0	1	IA
**76**	ec	5.0	30.0	90.0	100.0	2	IA
**77**	ec	90.0	100.0	NA	NA	1	IB

**78**	ec	90.0	100.0	100.0	100.0	1	IA
**79**	ec	30.0	20.0	100.0	100.0	3	IA
**80**	ec	30.0	10.0	NA	NA	1	IB
**81**	ec	25.0	85.0	100.0	100.0	2	IIIA
**82**	ec	25.0	35.0	NA	NA	1	IA
**83**	ec	90.0	80.0	100.0	100.0	2	IA
**84**	ec	100.0	5.0	100.0	50.0	1	IIIA
**85**	ec	100.0	90.0	100.0	50.0	2	NA
**86**	ec	90.0	10.0	100.0	10.0	1	IA
**87**	ec	80.0	25.0	NA	NA	3	IB
**88**	ec	100.0	85.0	100.0	100.0	1	IA
**89**	ec	100.0	85.0	NA	NA	1	IA
**90**	ec	100.0	100.0	NA	NA	2	IIIB
**91**	ec	100.0	100.0	90.0	100.0	1	IA
**92**	ec	100.0	25.0	NA	NA	1	NA
**93**	ec	90.0	25.0	100.0	100.0	1	IA
**94**	ec	15.0	10.0	100.0	100.0	1	IA
**95**	ec	100.0	100.0	100.0	100.0	1	IB
**96**	ec	100.0	10.0	NA	NA	1	IA
**97**	ec	90.0	90.0	100.0	100.0	1	IB
**98**	ec	100.0	100.0	100.0	100.0	1	IA
**99**	ec	85.0	10.0	100.0	100.0	2	IA
**100**	ec	100.0	10.0	100.0	100.0	3	IB
**101**	ec	80.0	15.0	NA	NA	1	IA
**102**	sc	100.0	95.0	100.0	100.0	NA	IA
**103**	sc	100.0	100.0	100.0	100.0	NA	IIIC
**104**	sc	100.0	100.0	100.0	100.0	NA	IA
**105**	sc	90.0	100.0	100.0	100.0	NA	IB

**106**	sc	40.0	95.0	NA	NA	NA	IB
**107**	sc	50.0	40.0	NA	NA	NA	III C1
**108**	sc	5.0	10.0	.0	50.0	NA	IA
**109**	sc	50.0	100.0	95.0	100.0	NA	IA
**110**	sc	40.0	40.0	NA	NA	NA	IB
**111**	sc	25.0	100.0	100.0	100.0	NA	IA
**112**	sc	100.0	95.0	100.0	100.0	NA	IVB
**113**	sc	5.0	10.0	100.0	100.0	NA	IVB

* Percentages of immunohistochemically positive positive tumor/epithelial cells.

** Serous endometrial carcinomas are by definition high grade tumors.

IHC: immunohistochemistry; ec: endometrioid endometrial cancer; sc: serous endometrial cancer;

AKR1B1: aldo-keto reductase family 1 member B1; AKR1B10: aldo-keto reductase family 1 member B10.

NA: not available.

Supplement Table S2. Survival data

Case	Event death	Event disease relapse	Survival (in years since initial diagnosis)	Time to relapse (in years since initial diagnosis)
**1**	no	no	17.60	16.93
**2**	no	no	17.30	16.66
**3**	no	no	17.00	16.34
**4**	no	no	16.80	16.16
**5**	no	no	16.70	16.07
**6**	no	no	16.60	15.96
**7**	no	no	16.50	15.84
**8**	yes	NA	14.60	NA
**9**	yes	NA	13.70	NA
**10**	yes	NA	9.80	NA
**11**	yes	NA	7.80	NA
**12**	yes	NA	7.80	NA
**13**	yes	NA	5.30	NA
**14**	yes	NA	4.00	NA
**15**	no	no	15.70	15.09
**16**	no	no	15.50	14.90
**17**	yes	yes	2.70	1.32
**18**	no	no	15.30	14.67
**19**	yes	NA	6.30	NA
**20**	no	no	15.10	14.46
**21**	yes	NA	13.40	NA
**22**	yes	NA	13.90	NA
**23**	no	no	15.10	14.45

**24**	no	no	13.50	12.82
**25**	yes	NA	10.80	NA
**26**	no	no	13.40	12.79
**27**	yes	NA	12.30	NA
**28**	no	no	12.40	11.78
**29**	no	no	11.80	11.16
**30**	no	no	11.80	11.14
**31**	no	no	11.70	11.03
**32**	yes	no	11.40	10.99
**33**	yes	no	11.40	10.96
**34**	no	no	11.60	10.95
**35**	no	no	11.60	10.91
**36**	no	no	11.50	10.86
**37**	yes	NA	9.40	NA
**38**	no	no	10.50	9.83
**39**	no	no	10.50	9.81
**40**	no	no	10.50	9.84
**41**	yes	NA	5.30	NA
**42**	no	no	10.30	9.67
**43**	no	no	10.20	9.57
**44**	no	no	10.20	9.54
**45**	no	no	10.10	9.50
**46**	no	no	7.90	7.30
**47**	no	no	7.90	7.28
**48**	no	no	8.00	7.35
**49**	no	no	7.90	7.26
**50**	no	no	7.90	7.30
**51**	no	no	7.90	7.21

**52**	no	no	7.90	7.23
**53**	no	no	7.90	7.21
**54**	no	no	7.90	7.21
**55**	no	no	7.70	7.07
**56**	no	no	7.70	7.02
**57**	no	no	7.60	6.95
**58**	no	no	7.60	6.99
**59**	yes	NA	5.30	NA
**60**	no	no	7.60	6.94
**61**	no	no	7.50	6.89
**62**	no	no	7.50	6.88
**63**	no	yes	7.40	5.27
**64**	no	no	7.40	6.78
**65**	no	no	7.40	6.75
**66**	no	no	7.40	6.77
**67**	no	no	7.40	6.74
**68**	no	no	7.30	6.69
**69**	no	no	7.20	6.59
**70**	no	no	7.20	6.52
**71**	no	no	7.20	6.51
**72**	no	no	7.10	6.49
**73**	no	no	7.10	6.49
**74**	no	no	7.10	6.43
**75**	no	no	7.10	6.44
**76**	no	no	6.70	6.03
**77**	no	no	6.70	6.01
**78**	no	no	6.60	5.99
**79**	no	no	6.60	5.97

**80**	yes	no	6.20	5.92
**81**	yes	NA	.80	NA
**82**	no	no	6.50	5.82
**83**	no	no	6.50	5.84
**84**	yes	yes	5.10	4.30
**85**	no	yes	6.50	2.72
**86**	no	no	6.30	5.63
**87**	yes	yes	.50	.31
**88**	no	no	6.10	5.49
**89**	no	no	6.20	5.55
**90**	no	no	6.30	5.66
**91**	no	no	6.10	5.49
**92**	no	no	6.10	5.49
**93**	no	no	6.00	5.38
**94**	no	no	6.10	5.44
**95**	no	no	6.10	5.44
**96**	no	no	6.10	5.41
**97**	no	no	5.80	5.11
**98**	no	no	5.50	4.85
**99**	no	no	5.50	4.84
**100**	no	no	5.50	4.85
**101**	no	no	5.40	4.80
**102**	yes	yes	15.60	.21
**103**	yes	yes	2.10	1.60
**104**	no	no	13.40	12.77
**105**	yes	NA	8.60	NA
**106**	yes	yes	1.00	.72
**107**	yes	yes	1.00	.85

**108**	no	no	10.20	9.55
**109**	no	no	7.60	6.95
**110**	yes	yes	1.00	.77
**111**	yes	yes	4.20	3.69
**112**	yes	yes	1.90	.90
**113**	yes	yes	.40	.32

NA: not available.

Supplement Table S3. Pathological data

Case	Metastasis in lymph nodes	Invasion in myometrium*	Lymphovascular invasion	Invasion in cervix	Invasion in parametria
**1**	no	1	yes	NA	NA
**2**	no	1	no	NA	NA
**3**	no	0	no	NA	NA
**4**	no	0	no	NA	NA
**5**	no	0	no	NA	NA
**6**	no	2	yes	NA	NA
**7**	yes	2	yes	NA	NA
**8**	no	2	no	NA	NA
**9**	no	1	yes	NA	NA
**10**	no	1	yes	NA	NA
**11**	no	1	yes	NA	NA
**12**	no	1	no	NA	NA
**13**	no	1	no	NA	NA
**14**	no	1	no	NA	NA
**15**	no	0	no	NA	NA
**16**	no	1	no	NA	no
**17**	no	2	yes	NA	NA
**18**	no	0	no	NA	NA
**19**	no	1	no	NA	NA
**20**	no	0	no	NA	NA
**21**	no	2	yes	NA	NA
**22**	no	2	no	NA	NA
**23**	no	2	no	NA	NA
**24**	no	0	no	NA	NA
**25**	no	1	yes	NA	NA

**26**	no	1	yes	NA	NA
**27**	no	1	no	NA	NA
**28**	no	1	no	NA	NA
**29**	no	1	no	NA	NA
**30**	no	1	no	NA	NA
**31**	no	2	yes	NA	NA
**32**	no	1	yes	NA	NA
**33**	no	1	yes	NA	NA
**34**	no	0	no	NA	NA
**35**	no	0	no	NA	NA
**36**	no	1	yes	NA	NA
**37**	no	0	no	NA	NA
**38**	no	0	no	NA	NA
**39**	no	1	no	NA	NA
**40**	no	1	no	NA	NA
**41**	no	1	no	NA	NA
**42**	no	0	no	NA	NA
**43**	no	1	yes	NA	no
**44**	no	1	no	NA	NA
**45**	no	2	yes	NA	NA
**46**	no	1	no	NA	NA
**47**	no	1	no	no	no
**48**	no	2	no	no	no
**49**	no	1	no	no	no
**50**	no	1	no	NA	NA
**51**	no	1	no	NA	NA
**52**	no	2	no	NA	no
**53**	no	1	no	NA	NA

**54**	no	1	no	no	no
**55**	no	1	no	NA	NA
**56**	no	1	no	NA	NA
**57**	no	1	no	NA	NA
**58**	no	1	no	NA	NA
**59**	yes	2	yes	yes	yes
**60**	no	0	no	no	no
**61**	no	0	no	NA	NA
**62**	no	0	no	no	NA
**63**	no	2	no	no	no
**64**	no	2	yes	NA	NA
**65**	no	1	no	NA	NA
**66**	yes	1	no	NA	NA
**67**	no	1	no	NA	NA
**68**	no	1	no	NA	NA
**69**	no	1	no	NA	NA
**70**	no	1	no	no	no
**71**	no	1	no	yes	no
**72**	no	1	no	no	no
**73**	no	1	no	NA	NA
**74**	no	0	no	NA	NA
**75**	no	0	no	NA	NA
**76**	no	1	no	NA	no
**77**	no	2	no	NA	NA
**78**	no	0	no	NA	no
**79**	no	1	no	NA	NA
**80**	no	2	yes	NA	NA
**81**	no	1	no	NA	no

**82**	no	1	no	yes	no
**83**	no	1	no	no	no
**84**	no	2	no	NA	no
**85**	no	2	no	yes	no
**86**	no	0	no	yes	no
**87**	yes	2	yes	NA	NA
**88**	no	0	no	yes	NA
**89**	no	1	no	NA	NA
**90**	no	2	yes	yes	yes
**91**	no	0	no	no	no
**92**	no	2	yes	NA	no
**93**	no	1	no	NA	no
**94**	no	1	no	NA	NA
**95**	no	2	no	NA	NA
**96**	no	1	no	no	no
**97**	no	2	no	NA	NA
**98**	no	0	no	NA	no
**99**	no	0	no	no	no
**100**	no	2	no	NA	NA
**101**	no	1	no	NA	NA
**102**	no	1	no	NA	no
**103**	yes	2	yes	NA	NA
**104**	no	1	no	NA	NA
**105**	no	2	yes	NA	NA
**106**	yes	1	yes	NA	NA
**107**	yes	2	yes	NA	NA
**108**	no	1	no	NA	NA
**109**	no	0	no	NA	NA

**110**	no	2	yes	NA	NA
**111**	no	1	yes	NA	NA
**112**	yes	1	yes	NA	NA
**113**	yes	2	yes	NA	yes

*Invasion in myometrium; value 0: no invasion in myometrium; value 1: < 50% of the myometrial thickness; value 2: > 50% of the myometrial thickness.

NA: not available.

Supplement Table S4. Clinical data 1

Case	Age of patient (years)	Body weight (kg)	Body height (cm)	BMI (body mass index)	Menopausal status*	Partus status (number of children)
**1**	51	NA	NA	NA	NA	NA
**2**	65	97	170	34	1	0
**3**	49	NA	NA	NA	NA	NA
**4**	41	130	168	46	0	1
**5**	53	79	167	28	1	1
**6**	59	68	165	25	1	1
**7**	54	63	155	26	1	1
**8**	72	95	167	34	1	1
**9**	63	NA	NA	NA	NA	NA
**10**	68	83	162	32	1	1
**11**	78	84	160	33	1	2
**12**	73	75	162	29	1	1
**13**	75	83	160	32	1	1
**14**	52	70	160	27	0	3
**15**	36	92	165	34	0	2
**16**	44	55	166	20	0	1
**17**	68	68	164	25	1	NA
**18**	53	65	168	23	0	0
**19**	72	100	167	36	1	1
**20**	54	51	160	20	0	2
**21**	68	82	165	30	1	0
**22**	76	85	NA	NA	1	1
**23**	56	104	165	38	1	2
**24**	61	88	169	31	1	2

**25**	78	69	NA	NA	1	2
**26**	63	75	154	32	1	3
**27**	80	82	170	28	1	2
**28**	49	88	165	32	0	1
**29**	70	119	158	48	1	1
**30**	73	100	170	35	1	2
**31**	74	73	155	30	1	3
**32**	74	130	163	49	1	3
**33**	50	86	NA	NA	NA	NA
**34**	54	95	158	38	1	4
**35**	43	110	157	45	0	2
**36**	68	87	158	35	1	2
**37**	82	90	165	33	1	2
**38**	58	102	165	38	1	1
**39**	66	67	162	26	1	2
**40**	66	93	164	35	1	1
**41**	71	67	156	28	1	3
**42**	44	79	165	29	0	2
**43**	44	60	170	21	0	2
**44**	71	80	165	29	1	2
**45**	64	97	165	36	1	2
**46**	75	75	163	28	1	4
**47**	62	85	157	35	1	3
**48**	61	94	169	33	0	0
**49**	48	125	162	48	0	2
**50**	72	90	168	32	1	2
**51**	59	91	163	34	1	2
**52**	70	72	166	26	1	1

**53**	63	95	168	34	1	2
**54**	60	100	165	37	1	2
**55**	55	84	158	34	1	2
**56**	62	71	150	32	1	2
**57**	72	65	164	24	1	2
**58**	58	81	159	32	1	0
**59**	57	79	163	30	1	2
**60**	46	67	174	22	0	0
**61**	67	56	165	21	1	2
**62**	76	110	158	44	1	1
**63**	63	100	163	38	1	2
**64**	68	74	161	29	1	2
**65**	80	60	156	25	1	2
**66**	62	100	163	38	1	2
**67**	63	107	162	41	1	3
**68**	58	79	160	31	1	3
**69**	64	80	163	30	1	2
**70**	66	90	165	33	1	2
**71**	73	99	158	40	1	0
**72**	68	60	143	29	1	0
**73**	56	58	164	22	1	2
**74**	56	76	167	27	1	1
**75**	75	79	164	29	1	2
**76**	53	107	155	45	1	2
**77**	69	91	154	38	1	0
**78**	52	68	166	25	1	2
**79**	71	65	162	25	1	3
**80**	88	80	170	28	1	1

**81**	58	78	163	29	1	2
**82**	50	120	164	45	1	2
**83**	69	107	153	46	1	1
**84**	77	72	153	31	1	2
**85**	70	NA	NA	NA	1	2
**86**	66	95	159	38	1	2
**87**	69	80	155	33	1	2
**88**	76	67	155	28	1	2
**89**	77	64	162	24	1	3
**90**	61	73	162	28	1	0
**91**	61	70	157	28	1	2
**92**	59	NA	NA	NA	NA	NA
**93**	65	68	158	27	1	2
**94**	62	76	150	34	1	3
**95**	73	82	168	29	1	2
**96**	70	63	163	24	1	1
**97**	74	115	167	41	1	0
**98**	58	78	159	31	1	2
**99**	58	78	163	29	1	2
**100**	68	64	155	27	1	1
**101**	60	98	163	37	1	2
**102**	49	75	164	28	1	3
**103**	76	65	165	24	1	2
**104**	70	80	165	29	1	2
**105**	73	65	162	25	1	0
**106**	58	60	176	19	1	2
**107**	75	60	158	24	1	1
**108**	47	94	NA	NA	0	2

**109**	62	79	162	30	1	2
**110**	71	50	153	21	1	0
**111**	64	65	165	24	1	3
**112**	62	83	150	37	1	NA
**113**	64	99	160	39	1	2

*Menopausal status; value 0: premenopausal patient; value 1: postmenopausal patient;
NA: not available.

Supplement Table S5. Clinical data 2

Case	Lymphadenectomy	R0 resection	Adjuvant radiotherapy	Adjuvant Chemotherapy
**1**	0	1	yes	0
**2**	pelvic	1	no	0
**3**	pelvic	1	yes	0
**4**	pelvic	1	no	0
**5**	pelvic	1	no	0
**6**	pelvic	1	yes	0
**7**	pelvic	1	yes	Paclitaxel/Carboplatin
**8**	pelvic	1	no	0
**9**	pelvic	1	yes	0
**10**	0	1	yes	0
**11**	0	1	yes	0
**12**	pelvic	1	no	0
**13**	pelvic	1	yes	0
**14**	pelvic	1	no	0
**15**	pelvic	1	no	0
**16**	pelvic	1	no	0
**17**	pelvic	1	no	0
**18**	pelvic	1	yes	0
**19**	pelvic	1	no	0
**20**	pelvic	1	no	0
**21**	pelvic	1	yes	0
**22**	pelvic	1	yes	0
**23**	pelvic	1	yes	0
**24**	pelvic	1	no	0
**25**	pelvic	1	yes	0

**26**	pelvic	1	yes	0
**27**	0	1	no	0
**28**	pelvic	1	yes	0
**29**	0	1	no	0
**30**	pelvic	1	no	0
**31**	pelvic	1	yes	0
**32**	pelvic	1	yes	0
**33**	pelvic	1	no	0
**34**	pelvic	1	no	0
**35**	pelvic	1	no	0
**36**	pelvic	1	yes	0
**37**	pelvic	1	no	0
**38**	pelvic	1	no	0
**39**	pelvic	1	no	0
**40**	pelvic	1	no	0
**41**	pelvic	1	no	0
**42**	pelvic	1	no	0
**43**	pelvic	1	yes	0
**44**	pelvic	1	yes	0
**45**	pelvic	1	yes	0
**46**	pelvic	1	no	0
**47**	pelvic	1	no	0
**48**	pelvic	1	no	0
**49**	pelvic	1	no	0
**50**	pelvic	1	no	0
**51**	pelvic	1	no	0
**52**	pelvic	1	yes	0
**53**	pelvic	1	no	0

**54**	pelvic	1	no	0
**55**	pelvic	1	no	0
**56**	pelvic	1	no	0
**57**	pelvic	1	no	0
**58**	pelvic	1	no	0
**59**	pelvic	1	yes	Paclitaxel/Carboplatin
**60**	pelvic	1	no	0
**61**	pelvic	1	no	0
**62**	pelvic	1	no	0
**63**	pelvic	1	yes	0
**64**	pelvic	1	no	0
**65**	pelvic	1	no	0
**66**	pelvic+para aortic	1	yes	Paclitaxel/Carboplatin
**67**	pelvic	1	no	0
**68**	pelvic+para aortic	1	no	0
**69**	pelvic	1	no	0
**70**	pelvic	1	no	0
**71**	pelvic	1	yes	0
**72**	pelvic	1	no	0
**73**	pelvic	1	no	0
**74**	pelvic	1	no	0
**75**	pelvic	1	no	0
**76**	pelvic	1	no	0
**77**	pelvic	1	no	0
**78**	pelvic	1	no	0
**79**	pelvic+para aortic	1	no	0

**80**	pelvic	1	no	0
**81**	pelvic	1	no	0
**82**	pelvic	1	no	0
**83**	0	1	no	0
**84**	0	1	yes	0
**85**	pelvic	1	yes	0
**86**	pelvic	1	no	0
**87**	pelvic	1	no	Paclitaxel/Carboplatin
**88**	pelvic	1	no	0
**89**	pelvic	1	no	0
**90**	0	0	yes	Paclitaxel/Carboplatin
**91**	pelvic	1	no	0
**92**	pelvic	1	no	0
**93**	pelvic	1	no	0
**94**	pelvic	1	no	0
**95**	pelvic	1	no	0
**96**	pelvic	1	no	0
**97**	pelvic	1	no	0
**98**	pelvic	1	no	0
**99**	pelvic+para aortic	1	no	0
**100**	pelvic	1	yes	0
**101**	pelvic	1	no	0
**102**	pelvic	0	yes	0
**103**	pelvic	0	yes	Carboplatin
**104**	pelvic	1	no	0
**105**	pelvic	1	yes	0
**106**	pelvic	1	yes	0

**107**	pelvic	0	no	Paclitaxel/Carboplatin
**108**	pelvic	1	no	0
**109**	pelvic	1	no	0
**110**	pelvic	1	yes	0
**111**	pelvic	1	no	0
**112**	pelvic	1	yes	Paclitaxel/Carboplatin
**113**	pelvic	0	no	0

Resection value 1: Resection R0 achieved (no gross or microscopic tumor remains).

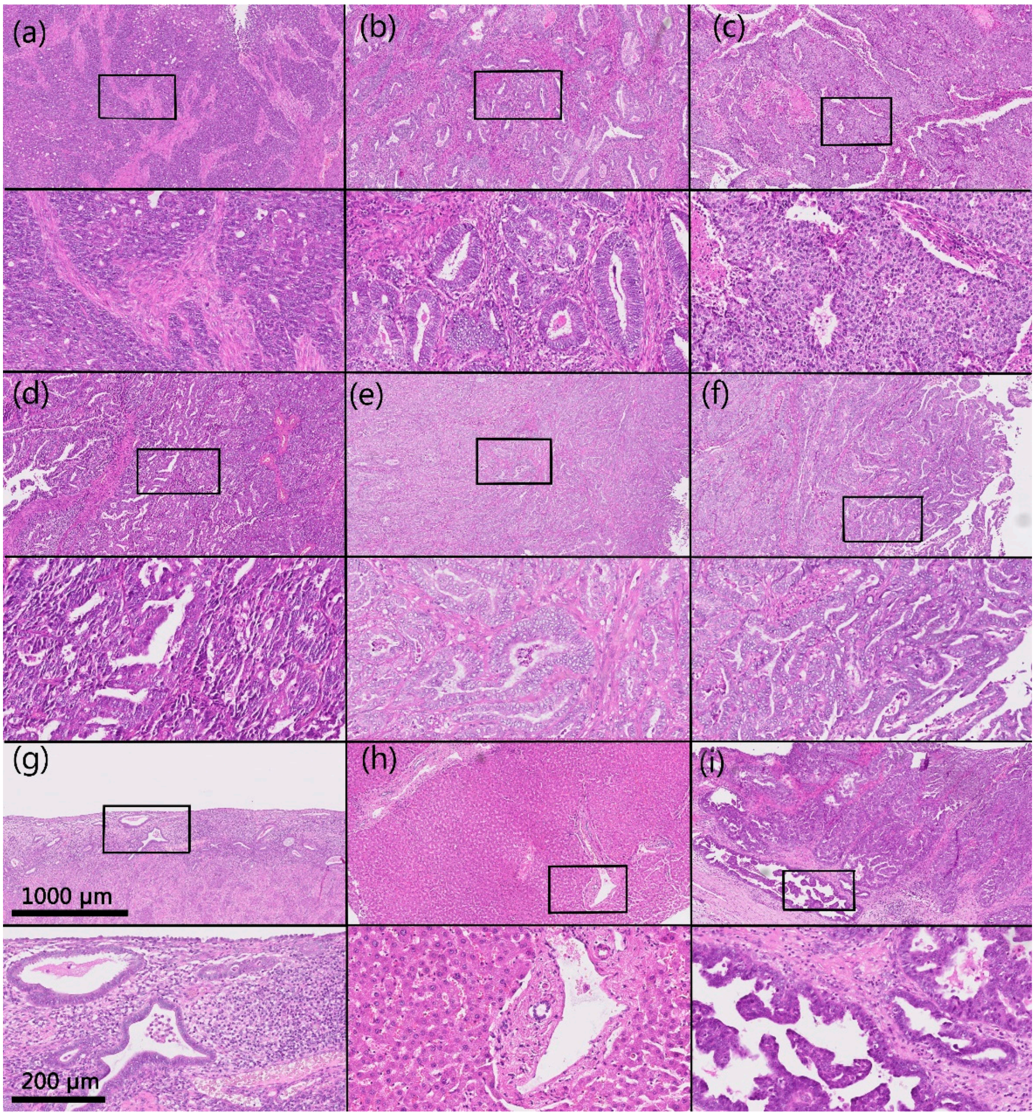

Supplementary Figure S1. Representative hematoxylin and eosin (HE) stained sections of the same samples as shown in Figure 2 in the main text of the manuscript.
Samples of endometrioid EC (a–c), serous EC (d–f), non-neoplastic endometrial tissue (g), control liver tissue (h) and control high grade serous ovarian cancer (i). Upper half of panels: 50× magnification; lower half of panels: the framed area from the upper half of the panel (200× magnification).

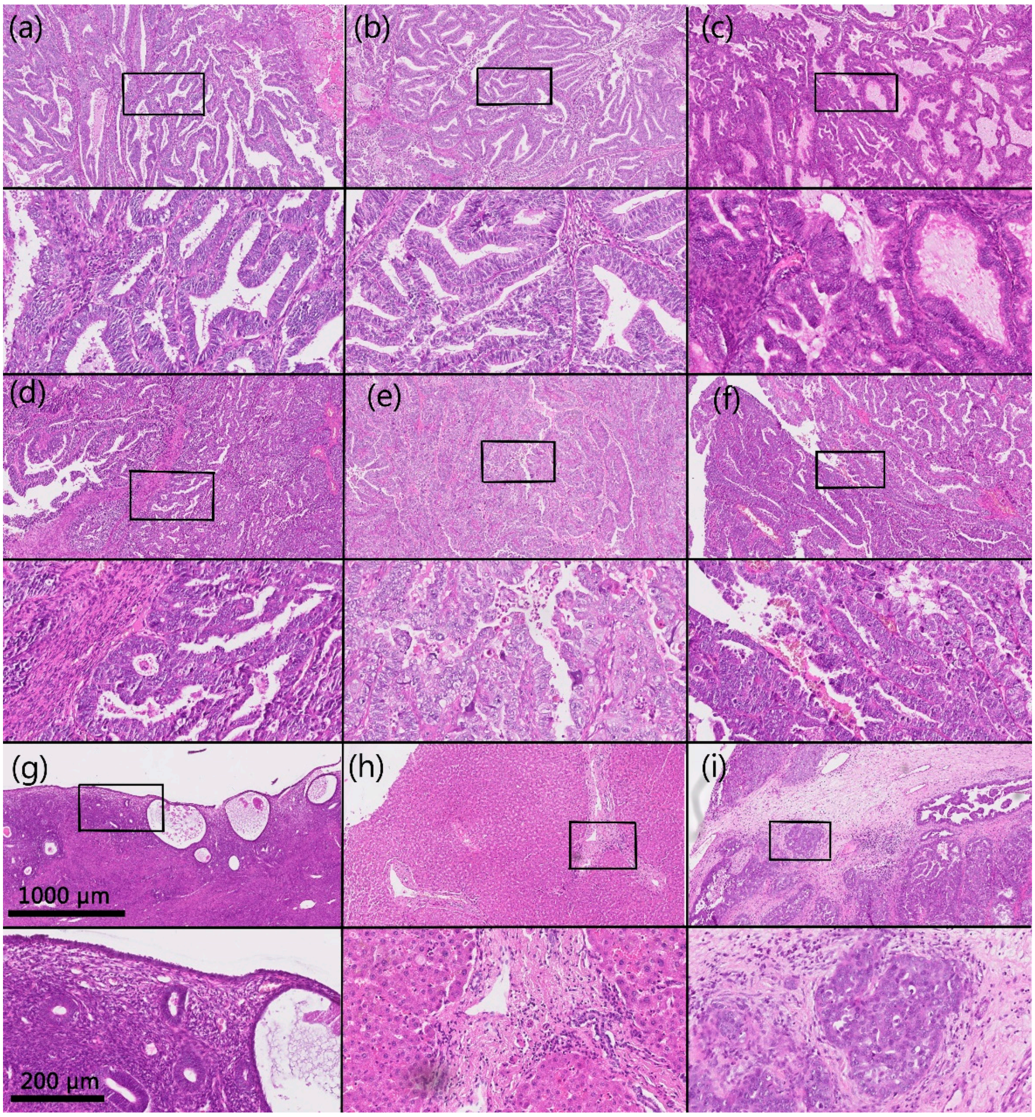

Supplementary Figure S2. Representative hematoxylin and eosin (HE) stained sections of same samples as shown in Figure 3 in the main text of the manuscript.

Samples of endometrioid EC (a–c), serous EC (d–f), non-neoplastic endometrial tissue (g), control liver tissue (h) and control high grade serous ovarian cancer (i). Upper half of panels: 50× magnification; lower half of panels: the framed area from the upper half of the panel (200× magnification).

Table S6. Survival analysis for low/ high risk endometrioid EC.

<u>**Endometrioid endometrial cancer**</u><br>**Grade 1-2 cases only,**<br>**Both AKR1B1 and AKR1B10 > median**			95,0% confidence interval	
	Significance	Hazard ratio	Lower	Upper
**Overall survival**	0.023	0.096	0.013	0.723
**Disease free survival**	0.022	0.180	0.042	0.780
**Grade 3 cases only,**<br>**Both AKR1B1 and AKR1B10 > median**				
**Overall survival**	0.683	1.518	0.204	11.276
**Disease free survival**	0.683	1.518	0.204	11.276

EC: endometrial cancer

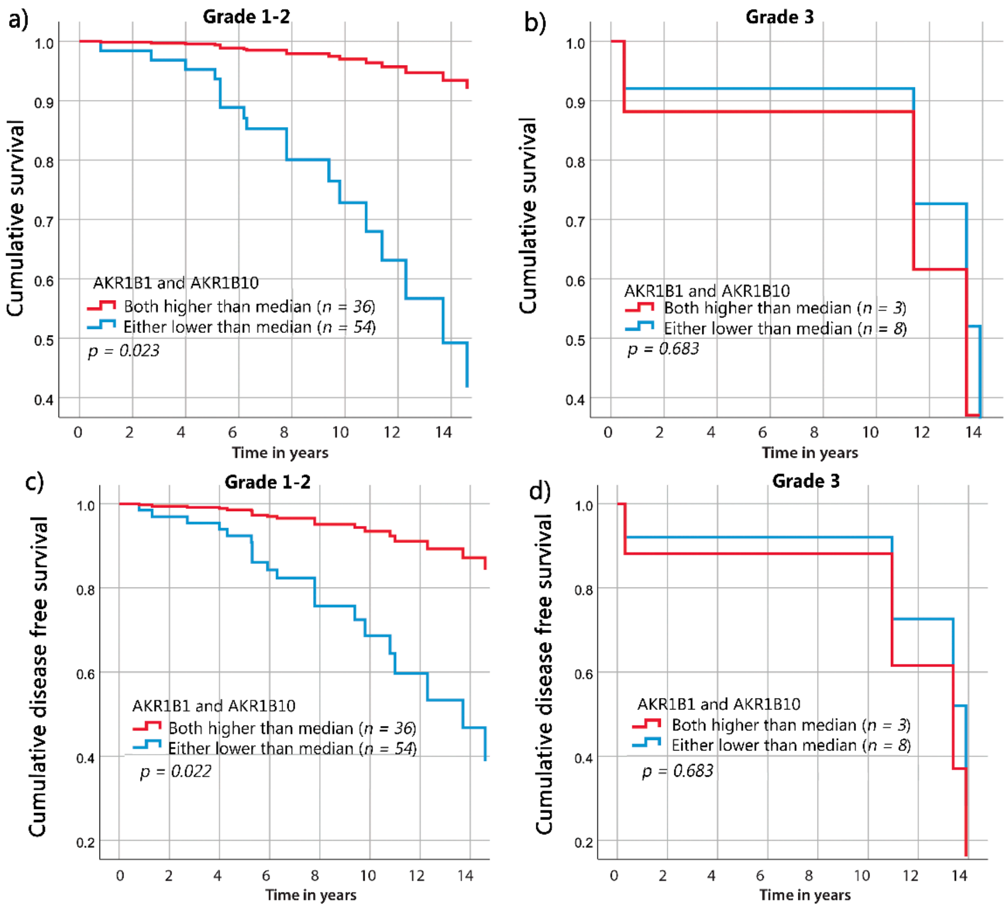

Supplementary Figure S3. Overall survival and disease-free survival in relation to AKR1B1 and AKR1B10 for patients with low/ high risk endometrioid EC.

One group includes cases with both AKR1B1 and AKR1B10 staining above the median values, and the other group includes cases with either AKR1B1 or AKR1B10 below the median values in endometrioid EC. Overall survival for endometrioid EC (**a**) grade 1-2 and (**b**) grade 3. Disease-free survival for endometrioid EC (**c**) grade 1-2 and (**d**) grade 3. Time on x axis represents time elapsed since initial diagnosis. AKR1B1: aldo-keto reductase family 1 member B1; AKR1B10: aldo-keto reductase family 1 member B10; EC: endometrial cancer.

Supplement Table S7. Survival analysis for FIGO I-II stage and for FIGO III-IV EC

Endometrioid endometrial cancer

**FIGO I-II stage cases only, Both AKR1B1 and AKR1B10 > median**			95,0% confidence interval	
	Significance	Hazard ratio	Lower	Upper
**Overall survival**	0.020	0.224	0.064	0.791
**Disease free survival**	0.014	0.209	0.060	0.729

**FIGO III-IV cases only, Both AKR1B1 and AKR1B10 > median**				
**Overall survival**	0.621	0.037	0.000	17396
**Disease free survival**	0.621	0.037	0.000	17396

EC: endometrial cancer

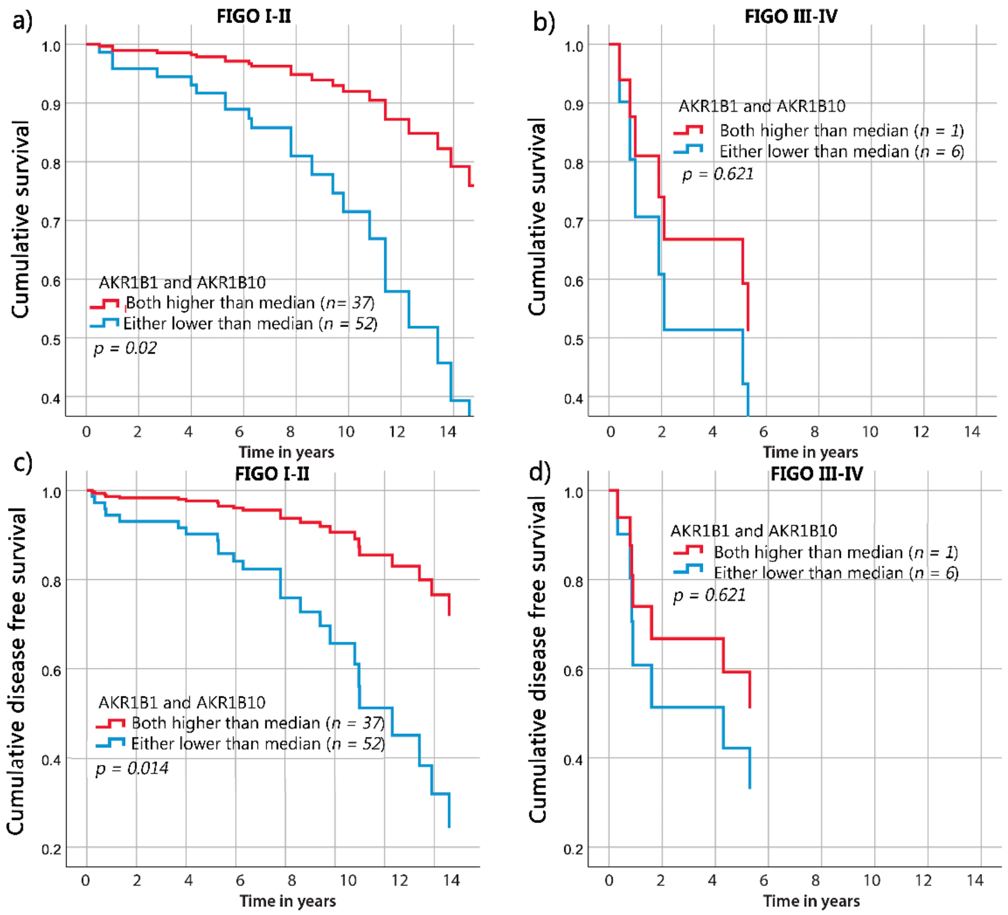

Supplementary Figure S4. Overall survival and disease-free survival in relation to AKR1B1 and AKR1B10 for patients with FIGO I-II/ FIGO III-IV disease.

One group includes cases with both AKR1B1 and AKR1B10 staining above the median values, and the other group includes cases with either AKR1B1 or AKR1B10 below the median values in endometrioid EC. Overall survival for endometrioid EC (**a**) FIGO I-II and (**b**) FIGO III-IV. Disease-free survival for endometrioid EC (**c**) FIGO I-II and (**d**) FIGO III-IV. Time on x axis represents time elapsed since initial diagnosis. AKR1B1: aldo-keto reductase family 1 member B1; AKR1B10: aldo-keto reductase family 1 member B10; EC: endometrial cancer.

Table S8. Associations between clinical and histopathological data and AKR1B1 and AKR1B10 immunohistochemical levels in patients with EC - part 1.

	AKR1B1 < median	AKR1B10 > median	AKR1B10 < median	AKR1B10 > median
Endometrioid EC				
Grade III	6/53 (11%)	5/48 (10%)	8/56 (14%)	3/45 (7%)
Lymphovascular invasion	14/53 (26%)	8/48 (17%)	12/56 (21%)	10/45 (22%)
Lymph node invasion	3/53 (6%)	1/48 (2%)	3/56 (5%)	1/45 (2%)
Cervical invasion	3/8 (38%)	4/12 (33%)	3/9 (33%)	4/11 (36%)
Parametrial invasion	1/12 (8%)	1/16 (6%)	0/14 (0%)	2/14 (14%)
Menopausal status	43/50 (86%)	40/46 (87%)	47/52 (90%)	36/44 (82%)
Parous status	43/53 (81%)	41/48 (85%)	45/56 (80%)	39/45 (87%)
Smoking status	4/28 (14%)	1/26 (4%)	5/32 (16%)	0/22 (0%)
Myometrial invasion	12/53 (23%)	12/48 (25%)	13/56 (23%)	11/45 (24%)
Weight (mean SD)	84.3 (16.6)	82.9 (18.1)	82.1 (14.7)	85.4 (19.8)
Height (mean SD)	162 (4.9)	161 (5.8)	162 (5.2)	163 (5.5)
BMI (mean SD)	32.0 (6.2)	31.9 (7.2)	31.6 (5.6)	32.4 (7.8)
Age (mean SD)	63.7 (9.7)	63.3 (10.9)	65.2 (9.3)	61.4 (11.1)
Serous EC				
Grade III	7/0 (100%)	5/0 (100%)	7/0 (100%)	5/0 (100%)
Lymphovascular invasion	5/7 (71%)	3/5 (60%)	5/7 (71%)	3/5 (60%)
Lymph node invasion	3/7 (43%)	2/5 (40%)	4/7 (57%)	1/5 (20%)
Parametrial invasion	1/1 (100%)	0/1 (0%)	1/2 (50%)	1/2 (50%)
Menopausal status	6/7 (86%)	5/5 (100%)	6/7 (86%)	5/5 (100%)
Parous status	6/7 (86%)	3/5 (60%)	5/7 (71%)	4/5 (80%)
Smoking status	4/0 (100%)	1/0 (100%)	3/0 (100%)	2/0 (100%)
Weight (mean SD)	72.4 (18.6)	73.6 (8.4)	74.4 (18.6)	70.8 (8.0)
Height (mean SD)	162 (7.8)	161 (6.4)	160 (9.2)	164 (1.6)
BMI (mean SD)	26.3 (7.1)	28.6 (5.2)	28.1 (8.1)	26.4 (3.1)
Age (mean SD)	63.0 (9.1)	66.0 (10.8)	60.9 (10.4)	69.0 (5.9)

None of the clinicopathological parameters differs statistically significantly between values <median or >median of AKR1B1 or AKR1B10. There were some missing data (cervical invasion, parametrial invasion, smoking).
EC: endometrioid caner; SD: standard deviation; BMI; body mass index; AKR1B1: aldo-keto reductase family 1 member B1; AKR1B10: aldo-keto reductase family 1 member B10.

Supplement Table S9. Associations between clinical and histopathological data and AKR1B1 and AKR1B10 immunohistochemical levels in patients with EC – part 2.

	Both AKR1B1 or AKR1B10 < median	Both AKR1B1 and AKR1B10 > median
Endometrioid EC		
Grade III	8/68 (12%)	3/33 (9%)
Lymphovascular invasion	17/68 (25%)	5/33 (15%)
Lymph node invasion	4/68 (6%)	0/33 (0%)
Cervical invasion	4/12 (33%)	3/8 (38%)
Parametrial invasion	1/19 (5%)	1/9 (11%)
Menopausal status	56/64 (88%)	27/32 (84%)
Parous status	57/68 (84%)	27/33 (82%)
Smoking status	5/37 (14%)	0/17 (0%)
Myometrial invasion	16/68 (24%)	8/33 (24%)
Weight (mean SD)	83.4 (16.0)	84.1 (19.7)
Height (mean SD)	162 (5.0)	162 (6.0)
BMI (mean SD)	31.9 (6.1)	32.1 (7.8)
Age (mean SD)	64.5 (9.7)	61.3 (11.2)
Serous EC		
Lymphovascular invasion	6/9 (67%)	2/3 (67%)
Lymph node invasion	4/9 (44%)	1/3 (33%)
Parametrial invasion	1/2 (50%)	1/2 (50%)
Menopausal status	8/9 (89%)	3/3 (100%)
Parous status	7/9 (78%)	2/3 (67%)
Smoking status	0/5 (0%)	0/0 (0%)
Weight (mean SD)	73.9 (16.5)	70.0 (8.7)
Height (mean SD)	161 (7.9)	164 (1.7)
BMI (mean SD)	27.8 (7.1)	26.0 (2.9)
Age (mean SD)	61.3 (9.1)	73.0 (3.0)

None of the clinicopathological parameters differs statistically significantly between values <median or >median of AKR1B1 or AKR1B10. There were some missing data (cervical invasion, parametrial invasion, smoking).
EC: endometrioid caner; SD: standard deviation; BMI; body mass index; AKR1B1: aldo-keto reductase family 1 member B1; AKR1B10: aldo-keto reductase family 1 member B10.
